# Identification of influential probe types in epigenetic predictions of human traits: implications for microarray design

**DOI:** 10.1186/s13148-022-01320-9

**Published:** 2022-08-10

**Authors:** Robert F. Hillary, Daniel L. McCartney, Allan F. McRae, Archie Campbell, Rosie M. Walker, Caroline Hayward, Steve Horvath, David J. Porteous, Kathryn L. Evans, Riccardo E. Marioni

**Affiliations:** 1 Centre for Genomic and Experimental Medicine, Institute of Genetics and Cancer, University of Edinburgh, Crewe Road South, Edinburgh, EH4 2XU UK; 2 Institute for Molecular Bioscience, The University of Queensland, Brisbane, 4072 Australia; 3 Centre for Clinical Brain Sciences, University of Edinburgh, Chancellor’s Building, 49 Little France Crescent, Edinburgh, EH16 4SB UK; 4 MRC Human Genetics Unit, Institute of Genetics and Cancer, University of Edinburgh, Edinburgh, EH4 2XU UK; 5 Department of Human Genetics, David Geffen School of Medicine, University of California Los Angeles, Los Angeles, CA 90095-7088 USA; 6 Department of Biostatistics, Fielding School of Public Health, University of California Los Angeles, Los Angeles, CA 90095-1772 USA

**Keywords:** DNA methylation, Prediction, Methylation QTLs, Complex traits, Ageing

## Abstract

**Background:**

CpG methylation levels can help to explain inter-individual differences in phenotypic traits. Few studies have explored whether identifying probe subsets based on their biological and statistical properties can maximise predictions whilst minimising array content. Variance component analyses and penalised regression (epigenetic predictors) were used to test the influence of (i) the number of probes considered, (ii) mean probe variability and (iii) methylation QTL status on the variance captured in eighteen traits by blood DNA methylation. Training and test samples comprised ≤ 4450 and ≤ 2578 unrelated individuals from Generation Scotland, respectively.

**Results:**

As the number of probes under consideration decreased, so too did the estimates from variance components and prediction analyses. Methylation QTL status and mean probe variability did not influence variance components. However, relative effect sizes were 15% larger for epigenetic predictors based on probes with known or reported methylation QTLs compared to probes without reported methylation QTLs. Relative effect sizes were 45% larger for predictors based on probes with mean Beta-values between 10 and 90% compared to those based on hypo- or hypermethylated probes (Beta-value ≤ 10% or ≥ 90%).

**Conclusions:**

Arrays with fewer probes could reduce costs, leading to increased sample sizes for analyses. Our results show that reducing array content can restrict prediction metrics and careful attention must be given to the biological and distribution properties of CpG probes in array content selection.

**Supplementary Information:**

The online version contains supplementary material available at 10.1186/s13148-022-01320-9.

## Background

DNA methylation (DNAm) involves the addition of methyl groups to the fifth carbon of cytosine bases, typically in the context of cytosine-guanine dinucleotides (CpG sites). There are approximately 28 million CpG sites across the human genome [1, 2], of which 60–80% are methylated [3]. Illumina DNAm arrays are popular technologies for profiling genome-wide DNAm. The probe content on these arrays has been selected by experts to optimise the balance between gene coverage and array size. The Infinium HumanMethylation 450K and HumanMethylationEPIC (EPIC) arrays cover 99% of RefSeq genes and contain probes that interrogate 485,577 and 863,904 CpG sites, respectively [4, 5].

There are two primary methods to quantify the amount of methylation at CpG sites interrogated by Infinium probes. First, the Beta-value (or B value) is a ratio of the methylated probe intensity to the overall measured intensity (sum of methylated and unmethylated probe intensities) [6, 7]. The Beta-value ranges from 0 to 100% where 100% implies complete methylation across all copies of the site in a given sample. Second, M values reflect the log2 ratio of methylated probe intensities versus unmethylated probe intensities. Positive M values mean that the site is likely more methylated than unmethylated in a given sample, and a value close to zero indicates that the site is equally methylated and unmethylated. It has been found that approximately 0.5% of Illumina probes show significantly different estimates for methylation intensities when measured by other methods, such as Methylation Capture bisulfite sequencing [8]. Here, we focus on the Beta-value (derived from Illumina arrays) as it has a simpler biological interpretation and therefore allows us to intuitively categorise probes into hypo- and hypermethylated sites (mean Beta-value ≤ 10% or ≥ 90% across individuals), which may reflect invariant probes.

Illumina DNAm data are routinely utilised in health outcomes research. First, the arrays are employed in association studies to uncover individual genomic loci associated with disease states and other phenotypes [9]. Second, the total array content (450K or EPIC) can be used to estimate the contribution of DNAm to inter-individual variability in human traits [10, 11]. Third, machine learning algorithms can be applied to DNAm data to identify weighted linear combinations of probes that predict numerous phenotypes, including chronological age, smoking status and body mass index [12–14].

Genetic, demographic and environmental factors contribute to inter-individual variability in CpG methylation [15]. Common genetic factors that correlate with CpG methylation are termed methylation quantitative trait loci (mQTLs) and explain on average 15% of the additive genetic variance of DNAm [16]. Variation in CpG methylation might also reflect technical artefacts, including heterogeneity in sample preparation and batch effects [17]. A large number of probes exhibit low levels of inter-individual variation in a given tissue, including blood [18–22]. Several methods have been proposed to remove sites that are non-variable in diverse tissue types. The methods include mixture modelling, principal component analyses and empirically derived data reduction strategies [23–25]. In the context of locus discovery, these methods reduce the severity of multiple testing correction and might improve power to detect epigenetic associations with phenotypes. However, it is unclear if low-variability CpG probes affect the amount of phenotypic variance captured by DNAm. There is also a lack of studies that examine the influence of probe intensity characteristics on DNAm-based predictors.

**Fig. 1:**
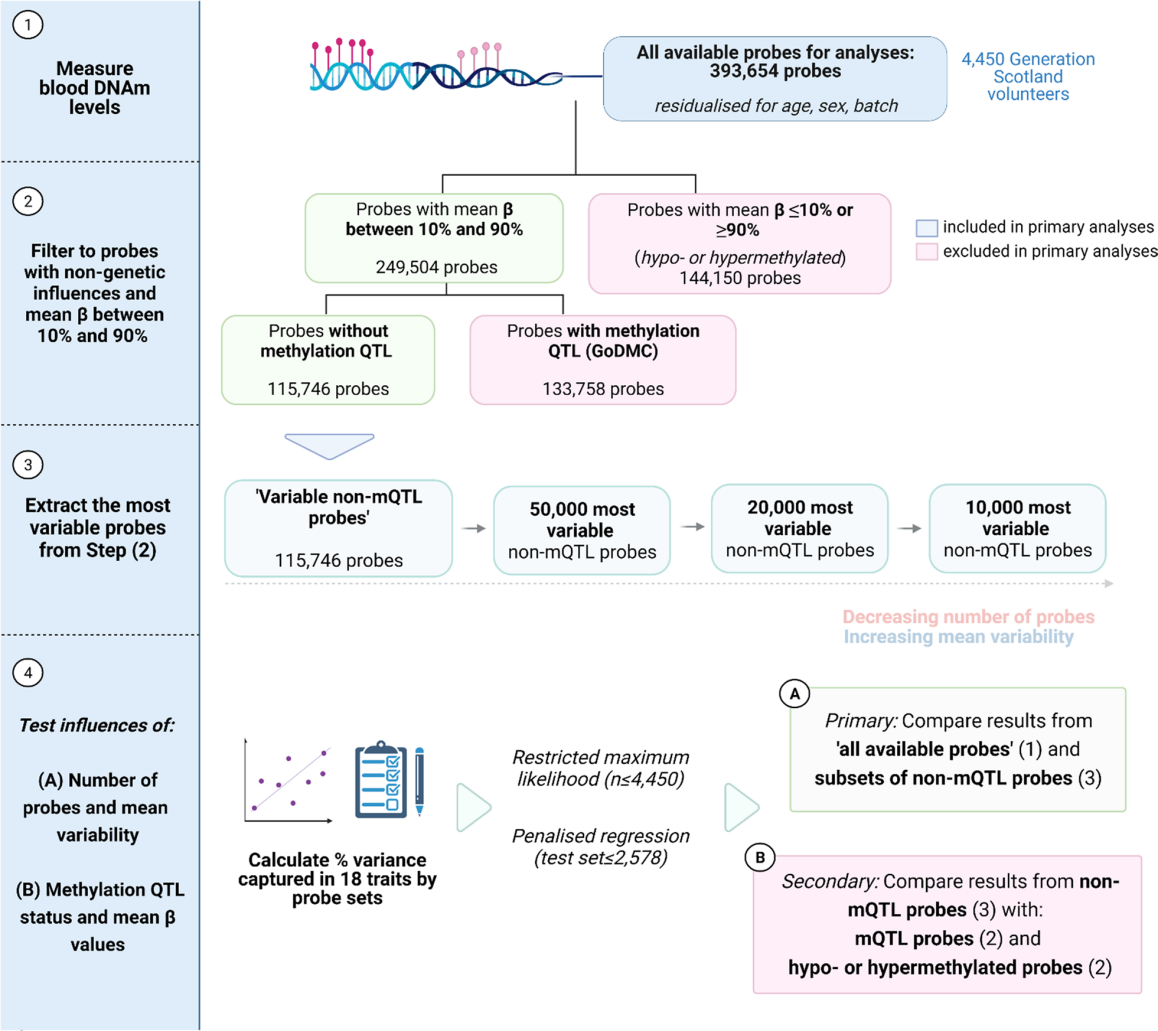
Overview of analysis strategy in the present study. We tested whether subsets of probes showed similar predictive capacities to total DNAm array content (1) (‘all available probes’, n = 393,654). We first identified subsets of interest. We restricted primary analyses to probes without known genetic influences (i.e. non-mQTL probes) and those with mean Beta-values (β) between 10 and 90% (2). These probes were termed ‘variable non-mQTL probes’ (n = 115,746). We then extracted the 50,000, 20,000 and 10,000 probes with the highest standard deviations from the pool of 115,746 non-mQTL probes (3). In our primary analyses, we compared the predictive performances of these four probe subsets against that of the full set of probes used in our analyses (4). In further analyses, we tested the relative performances of subsets based on (i) probes without known mQTLs and with mean Beta-value between 10 and 90% (shown in green in (2), highlighted in (3)), (ii) probes with known mQTLs and with mean Beta-value between 10 and 90% (shown in red in (2)) and hypo- or hypermethylated probes (mean Beta-value ≤ 10% or ≥ 90%, also shown in red in (2)). DNAm, DNA methylation; mQTL, methylation quantitative trait locus; SD, standard deviation. Image created using Biorender.com

Probes with high inter-individual variation in DNA methylation might be more informative for capturing variance in human traits compared to those that are invariant (i.e. low inter-individual variation). Here, we tested the hypothesis that invariant probes do not influence the amount of variance in phenotypes captured by Illumina array content. We utilised blood DNAm data and eighteen phenotypes from 4450 unrelated volunteers in the population-based cohort Generation Scotland as our training sample [26, 27]. We compared the performance of five primary sets of probes. The first set of probes, or the reference set, included all probes common to the 450K and EPIC arrays (n = 393,654 probes). We focussed on probes common to both arrays rather than focussing on the EPIC array alone in order to ensure generalisability to other and older cohort studies, which employ the 450K array. In the second set, we excluded hypo- and hypermethylated probes (e.g. mean Beta-value ≤ 10% or ≥ 90% across individuals). We also removed probes with mQTLs reported in the largest genome-wide association study on blood CpG methylation to date [16]. We employed these exclusion criteria in an effort to retain variable probes whose variability might largely reflect environmental contributions (n = 115,746 probes). The third, fourth and fifth sets included the 50,000, 20,000 and 10,000 most variable probes (i.e. highest standard deviations) without known mQTLs (Fig. 1).

**Fig. 2:**
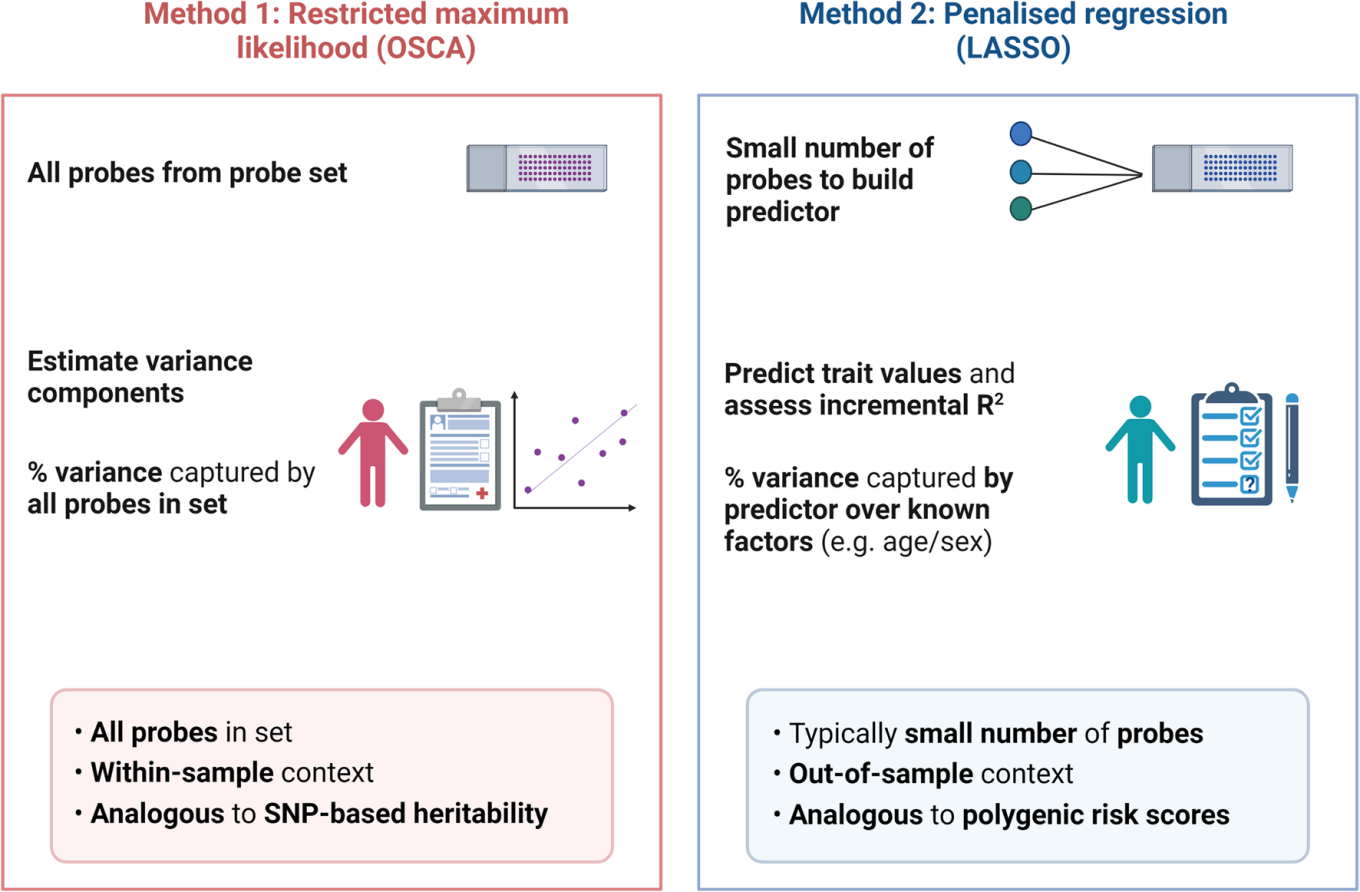
Distinction between two primary analysis methods in the present study. We employed both variance components and penalised regression models in order to examine the amount of phenotypic variance captured by each respective probe set (n = 18 in total, see Methods). Variance component estimates were obtained using the restricted maximum likelihood method in OSCA. Here, we were able to estimate the amount of phenotypic variance captured by all probes in a given probe set in the training sample (n ≤ 4450). We also employed penalised regression to build linear DNAm-based predictors of traits using probes in a given probe set in the training sample. We then applied the predictors to the test sample (n ≤ 2578) in order to estimate how much variance in a given trait the predictor could explain over basic covariates (such as age and sex). This coefficient reflected the incremental R^2^ estimate and pertained to an out-of-sample setting as the predictor was applied to a sample outside of that in which it was derived. LASSO, least absolute shrinkage and selection operator; OSCA, OmicS data-based complex trait analysis. Image created using Biorender.com

We used two methods to investigate how the number of probes considered in a probe set and how their distribution properties influenced the amount of phenotypic variance captured by DNAm. First, we estimated the amount of phenotypic variation captured by DNAm in the training sample (reflecting within-sample trait variance). For this, we used OmicS-data-based complex trait analysis (OSCA) software in which the correlation structure among all input probes is used to create an omic-data-based relationship matrix (ORM). The ORM is then used to estimate variance components through the restricted maximum likelihood method (REML) [10]. In essence, these estimates represent an upper bound of trait variance captured by DNAm in a given sample. Second, we applied penalised regression models to build DNAm-based predictors of all eighteen traits in the training sample. In DNAm prediction analyses, the substantially higher number of probes on arrays (features) when compared to observations (individual phenotype values) can lead to overfitting. For example, a predictor may perform well in the training data set but not in an external, independent data set. DNAm-based predictors derived from LASSO or elastic net penalised regression models often only consider small numbers of probes (derived from all input probes) to avoid such overfitting. The variances explained by these predictors are also smaller than those from REML, reflecting disparate methodologies and analysis objectives. In summary, REML utilises all input probes and estimates within-sample phenotypic variance and penalised regression considers a small subset of these probes to estimate the amount of variance captured by DNAm in out-of-sample settings (Fig. 2).

We compared results from the five primary sets of probes to test our primary hypothesis, and these probe sets had decreasing numbers of probes and increasing mean variabilities. In further analyses, we also considered secondary subsets of probes with (i) an mQTL (with a mean Beta-value between 10 and 90%), (ii) hypo- or hypermethylated probes (with a mean Beta-value ≤ 10% or ≥ 90%) and (iii) genome-wide significant EWAS Catalog probes (at *P* < 3.6 × 10^–8^). By comparing results from the primary and secondary probe sets, we were able to test the influence of (i) the number of probes considered, (ii) mean probe variability and (iii) methylation QTL status on the variance captured in eighteen traits by blood DNA methylation. Further, we compared results from these probe sets against those from randomly sampled sets of probes of equal size in order to determine whether observed estimates were significantly different from those expected by chance.

## Results

Demographics and summary data for all phenotypes are shown in Additional file 1: Table S1. The phenotypes were chronological age, seven biochemical traits (creatinine, glucose, high-density lipoprotein cholesterol, potassium, sodium, total cholesterol and urea) and ten complex traits (body fat percentage, body mass index, diastolic blood pressure, forced expiratory volume in one second (FEV), forced vital capacity (FVC), heart rate (average beats/minute), self-reported alcohol consumption, smoking pack years, systolic blood pressure and waist-to-hip ratio). The mean age in the training sample was 50.0 years (SD = 12.5), and the sample was 61.4% female. The test sample showed a similar mean age of 51.4 years (SD = 13.2) with a slightly lower proportion of females (56.3%). Values for all other phenotypes were comparable between the training and test samples.

### Phenotypic variance captured by DNAm decreases with the number of probes considered

We compared variance component estimates from ‘all available probes’ (n_probe_ = 393,654) and four subsets of probes with decreasing sizes and increasing mean variabilities (see Methods, Fig. 1). The subsets contained probes with mean Beta-values between 10 and 90% and without underlying mQTLs as reported by the GoDMC mQTL consortium (i.e. were non-mQTL probes) [16]. The first of these four subsets contained 115,746 probes, which represented all probes without reported mQTLs and with mean Beta-values between 10 and 90% (i.e. ‘variable non-mQTL probes’). The remaining three probe subsets harboured the 50,000, 20,000 and 10,000 most variable of the non-mQTL probes, showing the highest standard deviations in the training sample (n = 4450).

The proportion of phenotypic variance captured by ‘all available probes’ (n_probe_ = 393,654) ranged from 23.7% (standard error (se) = 6.0%) for blood potassium levels to 79.6% (se = 2.1%) for smoking pack years (Additional file 1: Table S2). The average proportion of variance captured across seventeen biochemical and complex traits was 54.0%. Mean estimates were 44.1% and 61.0% for biochemical and complex traits, respectively (Additional file 2: Fig. S1).

**Fig. 3:**
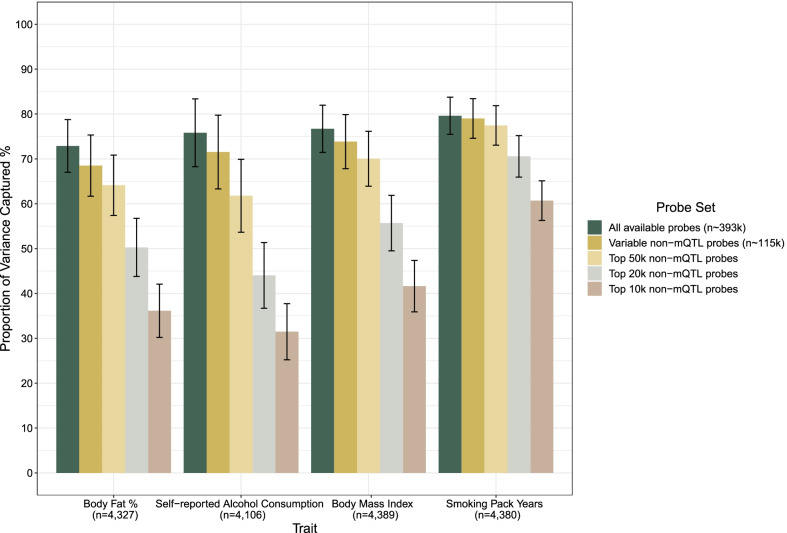
Variance captured in complex traits by all available probes and four subsets of decreasing size. Restricted maximum likelihood was used to estimate variance components in the training sample (n ≤ 4450, OSCA software). The four traits (out of seventeen biochemical and complex traits) with the highest proportion of variance captured by DNAm are shown. Five different sets of probes were compared. ‘All available probes’ denotes probes that were common to the Illumina EPIC and 450K arrays and passed quality control procedures in the training sample within Generation Scotland (n = 393,654 probes). The ‘variable non-mQTL probes’ set consisted of probes without reported non-genetic influences and mean Beta-values between 10 and 90%. The remaining three probe subsets contained the 50,000, 20,000 and 10,000 most variable non-mQTL probes (ranked by their standard deviations). The five sets of probes therefore had decreasing numbers of probes but increasing mean variabilities. Vertical bars show 95% confidence intervals. DNAm, DNA methylation; mQTL, methylation quantitative trait locus; OSCA, OmicS data-based complex trait analysis

The four remaining probe sets containing 115,746, 50,000, 20,000 and 10,000 probes, on average, captured 47.9%, 40.6%, 30.4% and 21.9% of phenotypic variance across seventeen traits, excluding chronological age (Additional file 1: Table S2). Generally, the estimates were not significantly different from sub-sampled probe sets of equal size, which were sampled from ‘all available probes’ (Additional file 1: Table S3). An exception to this was smoking pack years (*P* < 0.05). Figure 3 shows the four traits with the highest proportion of phenotypic variance captured by probe values.

### Performance of DNAm-based predictors decreases with the number of probes considered

DNAm-based predictors based on ‘all available probes’ (n_probe_ = 393,654) captured between 0.74% (forced vital capacity) and 46.0% (smoking pack years) of trait variance in the test sample (Additional file 1: Table S4). DNAm-based predictors developed from ‘all available probes’ on average captured 9.1% of trait variance (Additional file 2: Fig. S2).

**Fig. 4:**
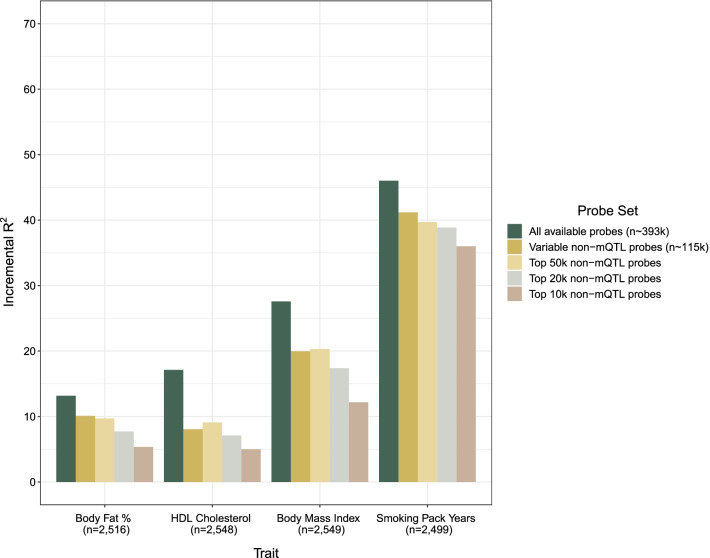
DNAm-based prediction of complex traits using all available probes and four subsets of decreasing size. LASSO regression was used to build blood DNAm-based predictors of seventeen biochemical and complex traits (n ≤ 4450 training sample and n ≤ 2578 test sample). The four traits with the highest proportion of variance captured by DNAm predictors in the test sample are displayed (incremental R^2^ estimates above null model, see main text). The first set of probes included those that passed quality control in the training sample, were common to both the EPIC and 450K arrays and included both probes with known methylation QTLs (mQTLs) and probes without known mQTLs reported in the GoDMC consortium. The next four sets of probes included non-mQTL probes only and had decreasing numbers of probes but increasing mean variabilities. DNAm, DNA methylation; HDL, high-density lipoprotein; LASSO, least absolute shrinkage and selection operator; mQTL, methylation quantitative trait locus

DNAm-based predictors developed from the four subsets of non-mQTL probes (in order of decreasing size) captured 6.7%, 6.6%, 5.6% and 5.0% of phenotypic variation. The four traits with the highest incremental R^2^ estimates are shown in Fig. 4.

The performances of the four subsets of non-mQTL probes were weaker for biochemical measures than complex traits. For biochemical measures, relative effect sizes were 19.1–38.7% of the magnitude of estimates from ‘all available probes’. The corresponding estimates were 47.5–74.2% for complex traits (Additional file 1: Table S4). Incremental R^2^ estimates were comparable to maximal R^2^ estimates from the literature achieved with similar, linear DNAm-based predictors. These analyses are distinct from the earlier variance component analyses and reflect the performance of DNAm-based predictors in samples external to those in which they were developed (Additional file 1: Table S5). Incremental R^2^ estimates from the four probe subsets were also not significantly different from sub-sampled sets of equal size (Additional file 1: Table S6).

### Subsets of probes capture similar amounts of variation in chronological age as total array content

Using REML, ‘all available probes’ captured 100% of variability in chronological age (n_probe_ = 393,654). Subsets that contained 115,746, 50,000 and 20,000 probes also captured 100% of the variance. The subset containing the 10,000 most variable non-mQTL probes captured only 92.1% (se = 0.9%, Additional file 1: Table S7).

An epigenetic age predictor based on ‘all available probes’ explained 91.7% of the variance in chronological age in the test sample (n = 2578). The R^2^ estimates from four subsets (in order of decreasing size) were 87.4%, 87.7%, 85.7% and 83.9%, respectively (Additional file 1: Table S8). The estimates were not significantly different from those in randomly sampled subsets with an equivalent number of loci.

### Highly variable probes are enriched for intergenic and upstream features

We tested whether the most variable set of probes, i.e. the 10,000 most variable non-mQTL probes, were over or under-represented for certain genomic features. We compared genomic annotations from this subset to annotations from 1000 sub-sampled sets of 10,000 probes, which were drawn from all available non-mQTL probes (n = 115,746, see Methods). Highly variable probes were enriched in intergenic sites, 5’UTR regions and sites lying 200–1,500 bases upstream from a transcription start site (range of fold enrichment (FE) = [1.1, 1.2], FDR-adjusted P = 0.001). They were also significantly under-represented within 3’UTR regions and gene bodies (FE = 0.8, *P* = 0.001; Additional file 1: Table S9).

### Methylation QTL status and mean probe variabilities do not influence variance component estimates

We performed further secondary analyses to determine the relative predictive capacities of four classes of probes. The first three classes were: (i) probes *without* a known mQTL and mean Beta-value between 10 and 90% (considered in primary analyses), (ii) probes *with* a known mQTL and mean Beta-value between 10 and 90% and (iii) probes with mean Beta-value ≤ 10% or ≥ 90%, that is, hypo- or hypermethylated probes (containing both mQTL and non-mQTL probes). The latter two classes are shown as the excluded probes in Fig. 1. We also considered a fourth class, which was EWAS Catalog probes (n = 38,853, see Methods). The EWAS Catalog probes contained all three of the other classes: > 65% were sites with an mQTL and < 5% were hypo- or hypermethylated (Additional file 1: Table S10).

Across all classes, variance estimates decreased with the number of probes under consideration (Table 1). All probe classes, when matched for the number of probes, showed comparable variance component estimates (Table 1; Additional file 1: Tables S11–S13). An exception to this involved subsets that included 115,746 probes. Probes with mean Beta-values between 10 and 90% on average captured 10% more trait variance than hypo- or hypermethylated probes (mean Beta-value ≤ 10% or ≥ 90%) at this threshold. The probe classes captured similar amounts of variance in age (Additional file 1: Table S14).

**Table 1:** Influences of the number of probes, mean variability and methylation QTL status on variance component estimates

Probe classification	Starting n_probe_	115,746	50,000	20,000	10,000
Probes without reported mQTL	115,746	47.9%	40.6%	30.4%	21.9%
Probes with reported mQTL	144,150	48.0%	38.7%	26.1%	16.5%
Hypo- and hypermethylated probes	133,758	38.7%	37.9%	27.7%	18.4%
EWAS Catalog probes	38,853	–	–	32.9%	24.4%

Metric shown is the average % of variance captured in seventeen biochemical and complex traits. mQTL, methylation QTL

### Probes with methylation QTLs and intermediate Beta-values are important for out-of-sample trait predictions

Epigenetic predictors based on EWAS Catalog probes (n = 38,853) captured as much variance as those based on ‘all available probes’ (n_probe_ = 393,654). The 20,000 and 10,000 most variable EWAS Catalog probes showed estimates that were 91.5% and 85.3% of the magnitude of those from all ‘available probes’ (Additional file 1: Tables S15–S17).

Epigenetic predictors based on probes with an mQTL (n = 133,758), and the 115,746 most variable of these probes, also captured as much phenotypic variance as predictors based on ‘all available probes’ (Additional file 1: Table S15). Exceptions included predictors for creatinine and systolic blood pressure (60–70% of estimates from ‘all available probes’).

The relative effect sizes (i.e. relative incremental R^2^ estimates) were on average 15% larger for probes with mQTLs versus those without GoDMC mQTLs. Relative effect sizes were also approximately 45% greater for probes with mean Beta-values between 10 and 90% when compared to hypo- or hypermethylated probes with mean Beta-values ≤ 10% or ≥ 90% (Table 2, Additional file 1: Tables S15–S17).

**Table 2:** Influences of the number of probes, mean variability and methylation QTL status on DNAm-based predictions

Probe classification	Starting n_probe_	115,746	50,000	20,000	10,000
Probes without known mQTL	115,746	6.7%	6.6%	5.6%	5.0%
Probes with known mQTL	144,150	9.0%	8.1%	6.2%	4.8%
Hypo- and hypermethylated probes	133,758	4.2%	4.0%	3.3%	2.8%
EWAS Catalog probes	38,853	–	–	8.7%	8.0%

Metric shown is the average % of variance captured in seventeen biochemical and complex traits by DNAm-based predictors. mQTL, methylation QTL

The performances of age predictors were comparable for all classes except hypo- and hypermethylated probes, which showed R^2^ estimates that were 5–10% lower than other probe classes (Additional file 1: Table S18).

## Discussion

The amount of phenotypic variance captured by DNAm decreased in all traits as the number of probes under consideration decreased. Further, variance component estimates were similar for subsets with and without reported genetic influences and subsets with and without hypo- and hypermethylated probes. The estimates were also comparable to sub-sampled subsets of equal size. Therefore, the number of probes considered is an important determinant of the amount of within-sample trait variance that can be captured by DNAm. Methylation QTL status and mean probe variabilities did not appear to impact variance component estimates. By contrast, epigenetic predictors based on probe subsets with mQTLs generally outperformed those that contained probes without GoDMC mQTLs. Similarly, probes that had mean Beta-values between 10 and 90% outperformed subsets that contained hypo- and hypermethylated probes in out-of-sample trait predictions. Therefore, methylation QTL status and mean Beta-values are important factors in the performance of epigenetic trait predictions. As with variance component analyses, decreasing the number of probes considered resulted in poorer performing epigenetic predictors.

Highly variable probes were enriched for intergenic sites, which is consistent with the existing literature [21, 28, 29]. However, the most variable probes that fall outside of CpG islands can be poorly captured by arrays [30]. The list of the most variable probes might show variation between epigenomic data sets given differences in normalisation methods and systematic differences in cohort profiles. We also did not correct for additional covariates, such as cell-type heterogeneity, which could lead to differences in estimates for probe variabilities. However, OSCA, or OmicS-data-based complex trait analysis, can account for unmeasured confounders and correlation structures between distal probes induced by confounders [10]. This is possible owing to the creation of an ORM, which describes the correlation structure between all input CpGs in a given data set. The ORM is then used to estimate the joint effects of all probes on the phenotype providing an estimate of the proportion of phenotypic variance captured by DNAm through restricted maximum likelihood. We selected standard deviations to measure variability in probe methylation levels. However, some probes may show non-normal distributions of Beta-values or multimodal distributions (such as probes with mQTLs). This complicates the general application of one measure of variability across all probes. Nevertheless, our results showed comprehensively that decreasing the number of available sites reduced variance estimates regardless of mQTL status or mean Beta-value.

As part of our primary and secondary analyses, we separated Illumina probes into those that have a genome-wide significant mQTL reported in the GoDMC mQTL database and those that do not have an mQTL reported in this list [16]. The GoDMC resource represents the largest, blood mQTL data set for Illumina probes. However, it must be acknowledged that it does not represent an exhaustive list of all possible mQTLs, whether acting in *cis* or in *trans*. Most probes are likely to have a genetic variance component but effect sizes for mQTLs vary substantially with most probes explaining less than 5–10% of inter-individual variation in DNAm [16, 31]. Future work is needed to filter probes by the proportion of variance explained by mQTLs in order to identify those probes with highly influential mQTLs. The impact of probes with strong genetic influences on epigenetic predictions should be examined and in cohorts of different ethnicities and clinical populations, which was not possible in the present study. Importantly, our strategy of stratifying probes by mQTL status replicates that of existing studies that examine the technical and distribution properties of Illumina probes. For instance, Sugden et al*.* also stratified probes into those with known mQTLs and those without mQTLs [31] and showed that probes indexed by mQTLs are more reliably measured than their non-mQTL counterparts [32]. The superior performance of epigenetic predictors from mQTL subsets compared to non-mQTL subsets in our study could reflect the higher measurement reliability of mQTL probes, and the exclusion of loci with strong biological signals in the predictors based on non-mQTL probes. As our data and findings were derived from whole blood, methodological insights into the role of Illumina probe types on variance analyses should only be used to guide future studies with whole blood samples.

High R^2^ estimates from subsets based on EWAS Catalog probes likely reflect contributions from all probe classes (i.e. probes with and without an mQTL and hypo- or hypermethylated sites) and that many of the traits considered in this study feature in the EWAS Catalog. Furthermore, traits with strong epigenetic correlates were the most robust to changes in probe classification or the number of probes considered. For instance, REML suggested that 20,000 probes were sufficient to capture 100% of inter-individual variation in chronological age. However, only 90% of the variance in age could be explained by subsets containing 10,000 probes. Previously, it has been shown that 100% of the variance in chronological age is captured by DNAm in Generation Scotland and the Systems Genomic of Parkinson’s Disease consortium [33]. Further, permutation testing suggested that these results did not reflect overestimation. The REML estimates are broadly analogous to chip-based heritability estimates in genetic analyses, reflecting how much variance in a trait can be explained by the omics measure in a given sample. By contrast, the aim of the penalised regression analyses was to generate linear combinations of probes that are informative for predicting age or other traits, which we applied to a separate but similar sample. Our incremental R^2^ estimates (~ 90%) are in line with, albeit lower than, those from existing epigenetic age indices, which employ additional steps to ensure highly accurate age predictors [14, 33, 34]. Here, our aim was to assess the influence of the number of probes and probe distribution properties on epigenetic predictions of age and seventeen lifestyle and biochemical traits. With respect to age, we show that (i) small subsets of probes can capture age-related changes in DNAm, (ii) DNAm-based age predictors are not strongly affected by mQTL status and (iii) probes that are hypo- or hypermethylated are less informative for predicting age than probes with Beta-values between 10 and 90%.

## Conclusions

Restricting DNAm array probes to the most variable sites could improve power in association studies whilst minimising array content. We show that this approach hampers variance component analyses and that phenotypes with strong epigenetic correlates are the most robust to changes in the number of available probes. Further, loci with an mQTL and with intermediate DNAm levels are central to epigenetic predictions of clinically relevant phenotypes. Our results provide methodological considerations towards the goal of selecting reduced array content from existing methylation microarrays, which can afford more cost-effective methylomic analyses in large-scale population biobanks. However, substituting or removing probes results in alterations to chip design and possibly the background physiochemical properties of the array. Therefore, it is not appropriate to assess the transferability of the present findings to other, related platforms. Nevertheless, our data demonstrate that strategies aiming to minimise arrays using fewer probes must carefully select CpG or probe content in order to maximise epigenetic predictions of human traits.

### Methods

#### Study cohort

Details of Generation Scotland (GS) have been described previously [26, 27]. GS is a family-based, genetic epidemiology cohort that consists of 24,084 volunteers. There were 5573 families with a median size of 3 members (interquartile range = 2–5 members, excluding 1400 singletons). Genome-wide DNAm was profiled using blood samples from GS baseline (2006–2011). DNAm was processed in two separate sets of 5200 (2016) and 4585 samples (2019) [35].

#### Preparation of DNA methylation data

DNAm was measured using the Infinium MethylationEPIC BeadChip at the Wellcome Clinical Research Facility, Western General Hospital, Edinburgh. Methylation typing in the first set (n = 5200) and the second set (n = 4585) was performed using 31 batches each. Full details on the processing of DNAm data are available in Additional file 3. Poor-performing and sex chromosome probes were excluded, leaving 760,943 and 758,332 probes in the first and second sets, respectively. Participants with unreliable self-report questionnaire data (self-reported 'yes' for all diseases in the questionnaire), saliva samples and possible XXY genotypes were excluded, leaving 5087 and 4450 samples in the first and second set, respectively. In the first set, there were 2578 unrelated individuals (common SNP GRM-based relatedness < 0.05). In the second set, all 4450 individuals were unrelated to one another. Individuals in the first set were also unrelated to those in the second set. The second set (profiled in 2019) was chosen for OSCA models and as the training sample in DNAm-based prediction analyses given its larger sample size (n = 4450). Unrelated individuals from the first set (profiled in 2016) formed the test sample in DNAm-based prediction analyses (n = 2578). Linear regression models were used to correct probe Beta-values for age, sex and batch effects separately within the training (n = 4450) and test samples (n = 2578).

#### Identification of variable probes in blood

There were 758,332 sites in the training sample (n = 4450) following quality control. First, we restricted sites to those that are common to the 450K and EPIC arrays to allow for generalisability to other epigenetic studies (n = 398,624 probes). We excluded loci that were predicted to cross-hybridise and those with polymorphisms at the target site, which can alter probe binding (n_probe_ = 4970 excluded, 393,654 remaining) [36, 37]. These 393,654 probes represented the reference set in our analyses, which we defined as ‘all available probes’.

We then defined a set of criteria to identify variable probes within blood tissue, specifically. First, we removed sites that are hypo- or hypermethylated in the sample (i.e. mean Beta-value ≤ 10% or ≥ 90%, respectively, n_probe_ = 144,150 excluded). Hypo- and hypermethylated sites had a mean SD of 0.01 (range = [0.002, 0.13]). Probes with mean Beta-values between 10 and 90% (n_probe_ = 249,504) had a mean SD of 0.03 (range = [0.008, 0.33]). Second, we excluded 133,758 probes that overlapped with known blood-based mQTLs (GoDMC [16], *P* value < 5 × 10^–8^). There were 115,746 remaining sites, which represented the ‘variable non-mQTL probes’ subset. We then extracted the 50,000, 20,000 and 10,000 non-mQTL probes with the highest SDs (mean SD = 0.04, 0.05, 0.06, respectively, Additional file 1: Table S10).

#### Preparation of phenotypic data

Eighteen traits were considered in our analyses. Full details on phenotype preparation are detailed in Additional file 3. The seventeen biochemical and complex traits (excluding chronological age) were trimmed for outliers (i.e. values that were ± 4 SDs away from the mean). Fifteen phenotypes (excluding FEV and FVC) were regressed on age, age-squared and sex. FEV and FVC were regressed on age, age-squared, sex and height (in cm). Correlation structures for raw (i.e. unadjusted) and residualised phenotypes are shown in Additional file 2: Fig. S3 and S4, respectively. For age models, DNAm and chronological age (in years) were unadjusted. Residualised phenotypes were entered as dependent variables in OSCA or penalised regression models.

#### Variance component analyses

OSCA software was used to estimate the proportion of phenotypic variance in eighteen traits captured by DNAm in the training sample (n ≤ 4450) [10]. In this method, an omic-data-based relationship matrix (ORM) describes the co-variance matrix between standardised probe values across all individuals in a given data set. Here, the ORM was derived from age-, sex- and batch-adjusted Illumina probe data and is fitted as a random effect component in mixed linear models. Phenotypes were pre-corrected for covariates as described in the previous section. Restricted maximum likelihood (REML) was applied to estimate the variance components, i.e. the amount of phenotypic variance captured by all DNAm probes used to build an ORM. We developed 18 ORMs in total reflecting all probe sets described: (i) one for ‘all available probes’, (ii) four for the ‘variable non-mQTL probe’ sets, (iii) five for ‘variable mQTL probe’ sets, (iv) five for hypo- and hypermethylated probe sets and (v) three for EWAS Catalog probes. The probe sets are outlined in full in Additional file 1: Table S10.

The variance component estimates are analogous, but not equivalent, to SNP-based heritability estimates [38, 39]. However, SNP-based heritability estimates have an inference of association through causality. The epigenetic variance component estimates could reflect both cause and consequence with respect to the phenotype and are not readily extended to other samples with different background characteristics. REML estimates served as important within-sample variance estimates in the present study, allowing us to assess the impact of the number of probes used to build an ORM, and their properties, on the amount of phenotypic variance captured by probe values. We then applied penalised regression models to build linear DNAm-based predictors of the phenotypes in the training sample. We carried out these analyses in order to assess the relative predictive performances of the probe sets when applied to a separate test sample (n < 2578), described below.

#### LASSO regression and prediction analyses

Least absolute shrinkage and selector operator (LASSO) regression was used to build DNAm-based predictors of eighteen phenotypes. The R package *biglasso* [40] was implemented and the training sample included ≤ 4450 participants. The mixing parameter (alpha) was set to 1 and tenfold cross-validation was applied. The model with the lambda value that corresponded to the minimum mean cross-validated error was selected. Epigenetic scores for traits were derived by applying coefficients from this model to corresponding probes in the test sample (n = 2578). This method takes into account the correlation structure between probes, but only selects a weighted additive combination of probes that are informative for predicting a given trait. Therefore, epigenetic predictors or methylation risk scores are broadly analogous to polygenic risk scores, which often show R^2^ estimates that fall far below SNP-based heritability estimates [41]. Here, our goal was to compare the relative predictive performances of probe sets in an out-of-sample context, distinct from the earlier approach of estimating variance components within the training sample alone.

Linear regression models were used to test for associations between DNAm-based predictors (i.e. epigenetic scores) for the eighteen traits and their corresponding phenotypic values in the test sample. The incremental r-squared (R^2^) was calculated by subtracting the R^2^ of the full model from that of the null model (shown below). For the FEV and FVC predictors, height was included as an additional covariate in both models. For the age predictors, the R^2^ value pertained to that of the epigenetic score without further covariates.

$$Null \, model:{\text{Phenotype }}\sim {\text{ chronological age }} + {\text{ sex}}$$

$$Full \, model:{\text{Phenotype }}\sim {\text{ chronological age }} + {\text{ sex }} + {\text{ epigenetic score}}$$

#### Sub-sampling analyses

We tested whether variance components and incremental R^2^ estimates from probe sets were significantly different from those expected by chance. For OSCA estimates, we generated 1,000 sub-samples of 115,746, 50,000, 20,000 and 10,000 probes (to match the primary subsets of non-mQTL probes tested in our analyses). The sub-sampled sets were sampled from ‘all available probes’ (n_probe_ = 393,654). We also generated 100 sub-samples of 115,746, 50,000, 20,000 and 10,000 probes, and not 1,000 sub-samples, for LASSO regression in order to lessen the computational burden.

We tested whether highly variable probes were significantly over-represented or under-represented for genomic and epigenomic annotations. Annotations were derived from the *IlluminaHumanMethylationEPICanno.ilm10b4.hg19* package in R [42]. Annotations for the most variable primary subset (i.e. 10,000 non-mQTL probes) were compared against 1,000 sub-samples of non-mQTL CpGs with an equal number of probes. Here, probes were sub-sampled from the ‘variable non-mQTL probes’ set (n_probe_ = 115,746) and not from ‘all available probes’ (n_probe_ = 393,654) as the latter contains probes with and without mQTLs, which show different genetic architectures [16].

#### Comparisons of methylation QTL status and mean Beta-values

In addition to non-mQTL subsets (with mean Beta-values between 10 and 90%), we tested two further classes of probes. First, we considered probes with a reported mQTL from GoDMC (*P* < 5 × 10^–8^) that had mean Beta-values between 10 and 90% (n_probe_ = 133,758) [16]. Second, we considered all hypo- or hypermethylated probes (Beta-value ≤ 10% or ≥ 90%, n_probe_ = 144,150). We tested the performances of the 115,746, 50,000, 20,000 and 10,000 most variable probes from each of these three classes.

We also repeated REML and LASSO regression using EWAS Catalog probes [43]. EWAS Catalog probes contained sites with an mQTL, sites without an mQTL and hypo- and hypermethylated sites. We restricted EWAS Catalog probes to those with *P* < 3.6 × 10^–8^ [44] and those reported in studies with sample sizes > 1000. We also excluded studies related to chronological age due to the very large number of sites implicated and alsothose in which Generation Scotland contributed to analyses. There were 100 studies that passed inclusion criteria with 47,093 unique probes. Of these, 38,853 probes overlapped with ‘all available probes’ used in our analyses (n_probe_ = 393,654). To allow for comparison to other subsets, the 20,000 and 10,000 most variable EWAS Catalog probes (n_probe_ = 38,853) were extracted.

## Supplementary Information

**Additional file 1: Table S1.** Demographic and summary data for Set 1 and Set 2 - Generation Scotland. **Table S2.** REML analyses - primary probe sets (non-mQTL probes). **Table S3.** REML analyses—permuted probe sets. **Table S4.** DNAm-based prediction analyses—primary probe sets (non-mQTL probes). **Table S5.** R2 estimates from linear DNAm-based predictors in the literature. **Table S6.** DNAm-based prediction analyses—permuted sets. **Table S7.** REML for chronological age. **Table S8.** DNAm-based prediction of chronological age. **Table S9**. Enrichment of genomic features in highly variable sites. **Table S10.** Description of probe sets compared in variance component and prediction analyses. **Table S11.** REML—mQTL probes vs. non-mQTL probes. **Table S12.** REML—EWAS Catalog probes vs. non-mQTL probes. **Table S13.** REML—hypo- and hypermethylated probes vs. non-mQTL probes. **Table S14.** REML for chronological age—sensitivity analyses. **Table S15.** DNAm-based prediction—mQTL probes vs. non-mQTL probes. **Table S16.** DNAm-based prediction—EWAS Catalog probes vs. non-mQTL probes. **Table S17.** DNAm-based prediction—hypo- and hypermethylated probes vs. non-mQTL probes. **Table S18.** DNAm-based prediction of chronological age—sensitivity analyses.

**Additional file 2: Figure S1.** Phenotypic variance captured by five nested sets of probes with decreasing numbers of probes and increasing mean variabilities. Restricted maximum likelihood analyses were performed using blood DNAm and phenotypic data from 4450 volunteers in the training sample of Generation Scotland. Seventeen biochemical and complex traits are shown. The seventeen traits are arranged into six groups (A–F). Vertical bars indicate 95% confidence intervals. Alc, self-reported alcohol consumption; bmi, body mass index; cholest, total cholesterol; dBP, diastolic blood pressure; DNAm, DNA methylation; fat, body fat percentage; FEV, forced expiratory volume in one second; FVC, forced vital capacity; HDL, high-density lipoprotein cholesterol; HR, heart rate; mQTL, methylation quantitative trait locus; PckYrs, smoking pack years; sBP, systolic blood pressure; whr, waist-to-hip ratio. **Figure S2.** Incremental R2 estimates for DNAm-based predictors of seventeen traits using five nested sets of probes with decreasing numbers of probes and increasing mean variabilities. LASSO regression was used to build DNAm-based predictors of seventeen traits using data from 4450 volunteers in the training sample within Generation Scotland. An unrelated sample of 2578 individuals in Generation Scotland served as the test set. The seventeen traits are arranged into six groups of three traits (A–F). Alc, self-reported alcohol consumption; bmi, body mass index; cholest, total cholesterol; dBP, diastolic blood pressure; DNAm, DNA methylation; fat, body fat percentage; FEV, forced expiratory volume in one second; FVC, forced vital capacity; HDL, high-density lipoprotein cholesterol; HR, heart rate; LASSO, least absolute shrinkage and selection operator; mQTL, methylation quantitative trait locus; PckYrs, smoking pack years; sBP, systolic blood pressure; whr, waist-to-hip ratio. **Figure S3.** Correlation structure between raw (i.e. unadjusted) phenotypes in the training and test samples within Generation Scotland. The training (A) and test samples (B) had 4450 and 2578 unrelated individuals, respectively. Alc, self-reported alcohol consumption; bmi, body mass index; cholest, total cholesterol; dBP, diastolic blood pressure; fat, body fat percentage; FEV, forced expiratory volume in one second; FVC, forced vital capacity; HDL, high-density lipoprotein cholesterol; HR, heart rate; PckYrs, smoking pack years; sBP, systolic blood pressure; whr, waist-to-hip ratio. **Figure S4.** Correlation structure between residualised phenotypes in the training and test samples within Generation Scotland. The training (A) and the test samples (B) had 4450 and 2578 unrelated individuals, respectively. Phenotypes were adjusted for chronological age and sex (and height for FEV and FVC). Age was not adjusted but is included for completeness of comparisons. Alc, self-reported alcohol consumption; bmi, body mass index; cholest, total cholesterol; dBP, diastolic blood pressure; fat, body fat percentage; FEV, forced expiratory volume in one second; FVC, forced vital capacity; HDL, high-density lipoprotein cholesterol; HR, heart rate; PckYrs, smoking pack years; sBP, systolic blood pressure; whr, waist-to-hip ratio.

**Additional file 3.** Supplementary methods.

## Data Availability

According to the terms of consent for Generation Scotland participants, access to data must be reviewed by the Generation Scotland Access Committee. Applications should be made to access@generationscotland.org.

## Code availability

All code is available at the following GitHub repository: https://github.com/robertfhillary/Biological_Statistical_Properties_CpGs_Epigenetic_Predictions.
