# Supplementary material for: Identification of influential probe types in epigenetic predictions of human traits: implications for microarray design
Source: Clin Epigenetics. 2022 Aug 10;14:100. doi: 10.1186/s13148-022-01320-9 (PMC9367152; doi:10.1186/s13148-022-01320-9)
Supplement: Supplementary file 2 — Additional file 2: Figure S1. Phenotypic variance captured by five nested sets of probes with decreasing numbers of probes and increasing mean variabilities. Restricted maximum likelihood analyses were performed using blood DNAm and phenotypic data from 4450 volunteers in the training sample of Generation Scotland. Seventeen biochemical and complex traits are shown. The seventeen traits are arranged into six groups (A–F). Vertical bars indicate 95% confidence intervals. Alc, self-reported alcohol consumption; bmi, body mass index; cholest, total cholesterol; dBP, diastolic blood pressure; DNAm, DNA methylation; fat, body fat percentage; FEV, forced expiratory volume in one second; FVC, forced vital capacity; HDL, high-density lipoprotein cholesterol; HR, heart rate; mQTL, methylation quantitative trait locus; PckYrs, smoking pack years; sBP, systolic blood pressure; whr, waist-to-hip ratio. Figure S2. Incremental R2 estimates for DNAm-based predictors of seventeen traits using five nested sets of probes with decreasing numbers of probes and increasing mean variabilities. LASSO regression was used to build DNAm-based predictors of seventeen traits using data from 4450 volunteers in the training sample within Generation Scotland. An unrelated sample of 2578 individuals in Generation Scotland served as the test set. The seventeen traits are arranged into six groups of three traits (A–F). Alc, self-reported alcohol consumption; bmi, body mass index; cholest, total cholesterol; dBP, diastolic blood pressure; DNAm, DNA methylation; fat, body fat percentage; FEV, forced expiratory volume in one second; FVC, forced vital capacity; HDL, high-density lipoprotein cholesterol; HR, heart rate; LASSO, least absolute shrinkage and selection operator; mQTL, methylation quantitative trait locus; PckYrs, smoking pack years; sBP, systolic blood pressure; whr, waist-to-hip ratio. Figure S3. Correlation structure between raw (i.e. unadjusted) phenotypes in the training and test samples [file 13148_2022_1320_MOESM2_ESM.docx]

**Additional file 2 - Supplementary Figures.** The following information pertains to Supplementary Figures for the manuscript ‘*Identification of influential probe types in epigenetic predictions of human traits: implications for microarray design’* by Hillary *et al.*

**Figure S1. Phenotypic variance captured by five nested sets of probes with decreasing numbers of probes and increasing mean variabilities.** Restricted maximum likelihood analyses were performed using blood DNAm and phenotypic data from 4,450 volunteers in the training sample of Generation Scotland. Seventeen biochemical and complex traits are shown. The seventeen traits are arranged into six groups (A – F). Vertical bars indicate 95% confidence intervals. Alc, self-reported alcohol consumption; bmi, body mass index; cholest, total cholesterol; dBP, diastolic blood pressure; DNAm, DNA methylation; fat, body fat percentage; FEV, forced expiratory volume in one second; FVC, forced vital capacity; HDL, high-density lipoprotein cholesterol; HR, heart rate; mQTL, methylation quantitative trait locus; PckYrs, smoking pack years; sBP, systolic blood pressure; whr, waist-to-hip ratio.

**Figure S2. Incremental R^2^ estimates for DNAm-based predictors of seventeen traits using five nested sets of probes with decreasing numbers of probes and increasing mean variabilities.** LASSO regression was used to build DNAm-based predictors of seventeen traits using data from 4,450 volunteers in the training sample within Generation Scotland. An unrelated sample of 2,578 individuals in Generation Scotland served as the test set. The seventeen traits are arranged into six groups of three traits (A – F). Alc, self-reported alcohol consumption; bmi, body mass index; cholest, total cholesterol; dBP, diastolic blood pressure; DNAm, DNA methylation; fat, body fat percentage; FEV, forced expiratory volume in one second; FVC, forced vital capacity; HDL, high-density lipoprotein cholesterol; HR, heart rate; LASSO, Least Absolute Shrinkage and Selection Operator; mQTL, methylation quantitative trait locus; PckYrs, smoking pack years; sBP, systolic blood pressure; whr, waist-to-hip ratio.

**Figure S3. Correlation structure between raw (i.e. unadjusted) phenotypes in the training and test samples within Generation Scotland.** The training (A) and test samples (B) had 4,450 and 2,578 unrelated individuals, respectively. Alc, self-reported alcohol consumption; bmi, body mass index; cholest, total cholesterol; dBP, diastolic blood pressure; fat, body fat percentage; FEV, forced expiratory volume in one second; FVC, forced vital capacity; HDL, high-density lipoprotein cholesterol; HR, heart rate; PckYrs, smoking pack years; sBP, systolic blood pressure; whr, waist-to-hip ratio.

**Figure S4. Correlation structure between residualised phenotypes in the training and test samples within Generation Scotland.** The training (A) and the test samples (B) had 4,450 and 2,578 unrelated individuals, respectively. Phenotypes were adjusted for chronological age and sex (and height for FEV and FVC). Age was not adjusted but is included for completeness of comparisons. Alc, self-reported alcohol consumption; bmi, body mass index; cholest, total cholesterol; dBP, diastolic blood pressure; fat, body fat percentage; FEV, forced expiratory volume in one second; FVC, forced vital capacity; HDL, high-density lipoprotein cholesterol; HR, heart rate; PckYrs, smoking pack years; sBP, systolic blood pressure; whr, waist-to-hip ratio.
